# Diagnosis of COVID-19: Considerations, controversies and challenges

**DOI:** 10.7196/AJTCCM.2020.v26i2.099

**Published:** 2020-04-21

**Authors:** K Dheda, S Jaumdally, M Davids, J-W Chang, P Gina, A Pooran, E Makambwa, A Esmail, E Vardas, W Preiser

**Affiliations:** 1 Centre for Lung Infection and Immunity, Division of Pulmonology, Department of Medicine and UCT Lung Institute and South African MRC/UCT Centre for the Study of Antimicrobial Resistance, University of Cape Town, South Africa; 2 Faculty of Infectious and Tropical Diseases, Department of Immunology and Infection, London School of Hygiene and Tropical Medicine, UK; 3 Lancet Laboratories, Johannesburg, South Africa; 4 Division of Medical Virology, Department Pathology, Faculty of Medicine and Health Sciences, Stellenbosch University, Cape Town, South Africa; 5 National Health Laboratory Service, Cape Town, South Africa

**Keywords:** COVID-19, SARS-CoV-2, Pneumonia, RT-PCR, Immunoassays, Diagnosis

## Abstract

Coronavirus disease 2019 (COVID-19) due to a novel virus, severe acute respiratory syndrome coronavirus 2 (SARS-CoV-2), is a global pandemic that has resulted in over 1.5 million confirmed cases and close to 100 000 deaths. In the majority of symptomatic cases, COVID-19 results in a mild disease predominantly characterised by upper respiratory tract symptoms. Reverse transcription polymerase chain reaction (RT-PCR) using a nasopharyngeal sample is the mainstay of diagnosis, but there is an ~30% false negative rate early in the disease and in patients with mild disease, and therefore repeat testing may be required. RT-PCR positivity can persist for several days after resolution of symptoms. IgM and IgG antibody responses become positive several days after the onset of symptoms, and robust antibody responses are detectable in the second week of illness. Antibody-based immunoassays have a limited role in the diagnosis of early symptomatic disease. However, their incremental benefit over RT-PCR in the first 2 weeks of illness is currently being clarified in ongoing studies. Such assays may be useful for surveillance purposes. However, their role in potentially selecting individuals who may benefit from vaccination, or as a biomarker identifying persons who could be redeployed into essential employment roles, is being investigated. Rapid antibody-based immunoassays that detect viral antigen in nasopharyngeal samples are being developed and evaluated.

## Background

Coronavirus disease 2019 (COVID-19) due to a novel virus, severe acute respiratory syndrome coronavirus 2 (SARS-CoV-2), is now a global pandemic. There are more than 1.5 million confirmed cases across almost every country in the world, and at the time of writing (early April 2020) there were close to 100 000 recorded deaths.^[1]^ The diagnosis of COVID-19 can be challenging, and as with any disease entity, a number of factors, including disease stage, disease prevalence, patient profile and sample type and quality, can influence diagnostic test performance. In this review we outline the performance outcomes of key tests used to diagnose COVID-19, and considerations that modulate performance. The safety of healthcare workers collecting samples, laboratory safety aspects and experimental approaches such as detection of volatile organic compounds in exhaled breath, mass spectrometry studies of different sample types, methods of signal amplification and utility of other novel approaches are not discussed here.

## Indications for testing

Country-specific indications and criteria for testing have evolved rapidly, and are being updated as information emerges and as the epidemic progresses. These recommendations have been guided by the phase of the epidemic and available resources. Generally speaking, testing for COVID-19 should currently be considered in anyone with symptoms of an acute respiratory tract infection (upper or lower) and with or without systemic symptoms such as fever, fatigue and myalgia.^[2–5]^ In mild disease, testing directs the need for self-isolation and identification of new cases through contact tracing and testing of contacts. As the epidemic progresses, and with forecasted limited testing capacity, testing may be directed to specific subgroups or those with enhanced risk of a poor outcome.

## The differential diagnosis

In the clinical setting, COVID-19 will form part of the differential diagnosis of any acute respiratory presentation, including infectious causes of pneumonia (e.g. bacterial, influenza, other viral pneumonia, pneumocystis pneumonia, tuberculosis (TB), etc.), acute exacerbations of asthma and chronic obstructive pulmonary disease (COPD), acute pulmonary embolism, cardiac failure and other conditions. Relevant investigations will depend on the clinical context, and will likely include pulmonary imaging, relevant laboratory investigations, blood cultures and interrogation of urine and/or lower respiratory tract specimens to rule in a viral, mycobacterial, fungal and/or bacterial cause. Clinical and laboratory parameters that may suggest viral infection include pyrexia, acute malaise and myalgia, and lymphopenia. C-reactive protein is unhelpful in distinguishing COVID-19 from other infections. Procalcitonin is elevated in severe COVID-19 and when there is secondary bacterial infection.^[6,7]^ In early disease low procalcitonin may distinguish COVID-19 from bacterial infections, but not from other viral diseases (data are awaited to confirm this supposition). In those with underlying asthma or COPD the presence of pulmonary infiltrates may favour a respiratory infection-related cause, though a cardiac cause must also be considered in the differential diagnosis.

## The biological sample of interest

The most common sample types sent for testing, usually by means of reverse transcription polymerase chain reaction (RT-PCR), are nasopharyngeal and oropharyngeal samples obtained using a swab, placed in viral transport medium. There is already considerable shortage of reagents (and swabs), meaning that dry swabs are being sent to laboratories in some centres. Dry swabs are less costly and more conducive to community-based testing, but data are urgently required to determine the comparative sensitivity of dry swabs compared with using viral transport medium (taking into account the time from sample acquisition to sample processing). Samples from the lower respiratory tract including sputum, tracheal aspirate, bronchial washings and bronchoalveolar lavage may also be sent. In patients with COVID-19 disease, samples from the lower respiratory tract are more likely to test positive (discussed below). Viral RNA can also be detected in stool in ~30% of cases, and in blood in ~1% of cases,^[8]^ but rarely in urine.

## Clinical and immuno diagnostic trajectory of COVID-19 and sampling considerations 

Recent data from infections in special contexts such as cruise liners^[9]^ and in close contacts of COVID-19 patients^[10]^ have demonstrated that SARS-CoV-2-specific RT-PCR may be positive in the early phase of the disease, and that viral shedding in the asymptomatic phase and in the early prodromal phase can be considerable.^[11,12]^ At present, screening of asymptomatic individuals by RT-PCR has been constrained by limited testing capacity, and the need to focus public health efforts and resources on symptomatic persons.

In symptomatic individuals, 80 - 90% of patients have mild symptoms not requiring hospitalisation. Depending on age and the presence of risk factors, ~10 - 20% of symptomatic persons may require admission to hospital because of respiratory or other complications. Individuals in this enhanced risk category may have one or more such factors, including age >50 years, comorbidities, history of significant tobacco smoking and underlying immunocompromising illnesses.^[10,13]^ In mild disease, especially in the early stages, the RT-PCR false negative rate is ~30 - 40%.^[8,14,15]^ A meta-analysis reported that a single test ~10 days post symptom development had an ~33% false negative rate using a nasopharyngeal swab (52.89% for a throat swab).^[16]^ Ai *et al*. ^[17]^ reported a false negative rate of 41% in a cohort of 1 014 hospitalised patients; the estimated median (standard deviation) interval between the initial negative test and subsequent positive RT-PCR result was 5.1 (1.5) days. A selection of other studies reported false negative rates of between 3 and 29%.^[18–20]^ Notably, some patients required up to five repeat tests before a positive result was ascertained.^[19]^ This false negativity phenomenon may be the result of several factors, including a low viral load below the detection limit of the assay, low sample volume or cellular mass during acquisition, sampling location (upper v. lower respiratory tract), sample degradation during transport or storage, sample processing methodology and the timing of sampling in relation to the stage of the disease (RT-PCR positivity may progressively increase during the course of the disease).^[14]^

Test accuracy will depend on the quality of the specimen collected.^[20]^ It has since been shown that specimens from the lower respiratory tract have a higher viral load, and are hence more likely to test positive than specimens from the upper respiratory tract.^[8,21]^ Nasopharyngeal specimens have better yield than oropharyngeal samples.^[8,15]^ In hospitalised patients with severe disease, Wang *et al*. ^[8]^ found a sensitivity of 93% in bronchoalveolar lavage fluid (a high aerosol-generating procedure), 72% in sputum and 63% in nasal swabs; sensitivity ranged from 0 to 32% in pharyngeal swabs, faeces, blood and urine. Given these considerations, a negative test from an upper respiratory tract specimen should be repeated after 1 - 3 days (the optimal timing is unclear), or a lower respiratory tract specimen obtained to exclude a false negative result, if clinical suspicion is high.^[22]^ Viral shedding in asymptomatic, early prodromal and minimally symptomatic individuals, and after resolution of symptoms, helps to explain the rapid and extensive spread of COVID-19. In patients with more severe disease, including those with lower respiratory tract infection, but also in individuals with mild disease, high viral loads can often be detected for several days after the resolution of symptoms.^[23]^ The significance of this remains unclear, although recent data from a limited number of patients suggest that RT-PCR positivity does not necessarily mean shedding of infectious virus after symptom resolution.^[11]^ Zhifeng *et al*. ^[24]^ demonstrated that RT-PCR using nasopharyngeal samples can be negative even when there is computed tomography scan evidence of COVID-19 pneumonia.^[24]^ Ali *et al*. ^[17]^ showed an improvement in disease extent in 42% of CT scans prior to RT-PCR tests becoming negative. It is unclear whether patients whose symptoms have resolved but who continue to have detectable viral RNA in respiratory samples can transmit infection. Furthermore, when symptoms have resolved, and especially given limited testing capacity, it remains unclear when patients may be discharged from the intensive care unit into the general ward setting, or from hospital into the community, especially if there are other individuals with high-risk profiles living in the same household. Therefore there are no clearly defined guidelines as to when it is safe for social mixing to occur after symptoms have resolved. Healthcare worker safety must be taken into account when collecting sputum, which should ideally be performed in an infection-controlled environment, or in the open air in ambulant patients.

## RT-PCR assays and their performance

**Fig. 1:**
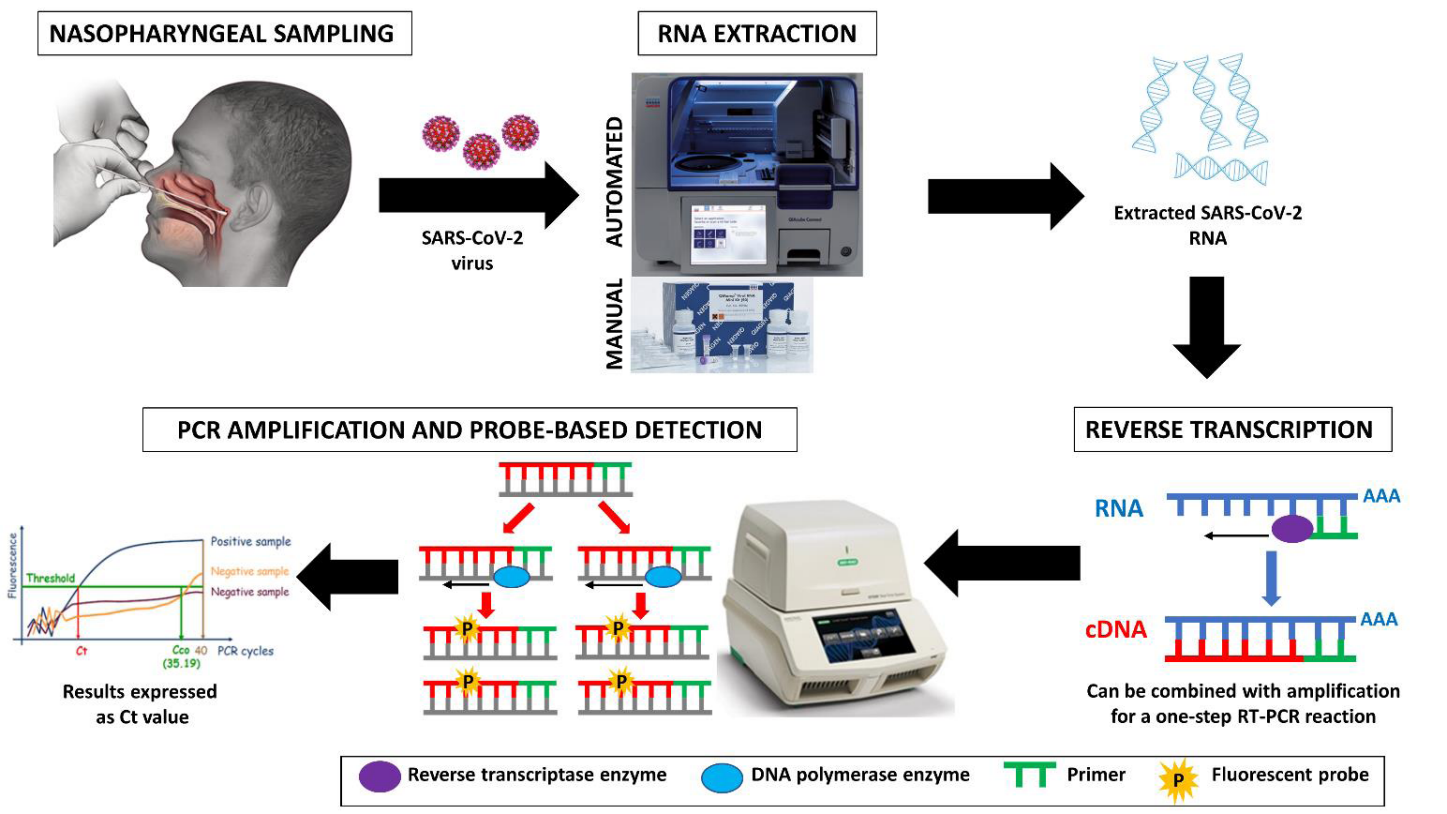
Overview of severe acute respiratory syndrome coronavirus 2 (SARS-CoV-2) reverse transcription polymerase chain reaction (RT-PCR)-based detection. Viral RNA extraction is performed from nasopharyngeal/oropharyngeal specimens using a manual or automated platform. RT-PCR is performed in a two-step assay. Extracted RNA is first reverse transcribed to make complementary DNA (cDNA). The cDNA is then amplified in the second step and fluorescence results from 5’-3’ exonuclease cleavage of a fluorescently-labelled target-specific probe enabling DNA amplification at each PCR cycle. (Ct = cycle threshold).

Currently, RT-PCR is the (imperfect) ‘gold standard’ for the diagnosis of COVID-19. The development of molecular detection assays has been facilitated by the sequencing of SARS-CoV-2.^[25]^ The assay consists of two principal steps: (i) viral RNA extraction from patient specimens performed manually or using automated platforms; and (ii) reverse transcription and PCR amplification using specific primers and specific probes for real-time detection (see Fig. 1 for an overview). The use of robotic systems allows for increased throughput for RNA extraction and PCR setup. Because of current resource constraints (trained personnel and reagents) and the necessity to rapidly deliver test outcomes, most diagnostic laboratories are skipping the post-extraction RNA quality and quantification check, which is costly and labour intensive.

The use of robotic systems allows for increased throughput for RNA extraction and PCR setup. Because of current resource constraints (trained personnel and reagents) and the necessity to rapidly deliver test outcomes, most diagnostic laboratories are skipping the postextraction RNA quality and quantification check, which is costly and labour intensive.

Several SARS-CoV-2 targets are being used, and these include the envelope, nucleocapsid and RNA-dependent RNA polymerase (RdRp) genes, and two large open reading frames orf1a/orf1b, and Ribonuclease P.^[4]^ Generally, at least two target genes need to be identified for SARS-CoV-2 confirmation. However, interpretation algorithms differ with respect to the number of genes that need to be detected for the test to be considered positive. For some protocols, results are interpreted as indeterminate or negative if one of the genes is not detected, whereas for others, identification of one gene is used as screening test, while that of the subsequent gene(s) serves as a confirmatory test.^[4]^ From a laboratory perspective, multiplexing of targets allows for better efficiency, shorter turnaround times and more optimal management of laboratory consumables.^[26]^ Vogels *et al*. ^[27]^ evaluated nine primer-probe sets. They confirmed that each pair had a detection efficiency of >90%, but there were differences in the ability to differentiate true negatives from positives in patients with a low viral load. Some sets led to inconclusive results due to nonspecific background amplification (including the initial sets issued by the US Centers for Disease Control and Prevention but with subsequent rectification). With viral evolution, nucleotide substitutions may emerge that could affect primer/probe binding regions that could alter the sensitivity of PCR. Indeed, a potentially problematic mismatch in the RdRp-SARSr reverse primer has already been confirmed. The threshold cycle value of the target gene remains the quantitative endpoint to ascertain viral load and, depending on the kit used, this value generally lies in the 30 - 40 range.^[4,27]^ To control for nonspecific PCR inhibition, an internal positive amplification control (e.g. SARS-CoV-2 E-gene RNA, SARS-CoV Frankfurt 1 RNA) is included in the assay, while a negative control interrogates for contamination during sample preparation.

Digital PCR (dPCR) has been used to perform a quality assurance verification of RT-PCR.^[28]^ dPCR involves partitioning a sample into many individual parallel PCR reactions, allowing even a single molecule to be amplified more than a million-fold. Using this technique, sensitivity was significantly improved from 28.2% by RT-PCR to 87.4% by RT-dPCR.^[28]^ Moreover, 15/16 close contacts in South Korea that were Guidelines for Laboratory Diagnosis of Coronavirus Disease 2019 (COVID-19)-inconclusive with conventional RT-PCR (likely because not all the targets of interest were detected) were dPCR-positive. The overall sensitivity, specificity and diagnostic accuracy of RT-dPCR was 90%, 100% and 93%, respectively. Moreover, the higher sensitivity of RT-dPCR translated into detection of viral RNA for longer periods than with conventional RT-PCR in convalescing patients. While RT-dPCR is more sensitive and suitable for detecting low viral loads, its accessibility is limited by the complexity of the system and cost implications, and the inability to multiplex target genes of interest.^[29]^

Several automated rapid nucleic acid amplification tests have recently received Food and Drug Administration (FDA) approval for emergency use. Cepheid’s Xpert Xpress SARS-CoV-2, run on the Gene Xpert platform, detects multiple gene targets and can provide a result within 45 minutes (https://www.cepheid.com/coronavirus). Abbott’s rapid COVID-19 test, run on the Abbott ID NOW device, can provide results within 13 minutes (https://www.alere.com/en/home/productdetails/id-now-covid-19.html). The former may be convenient in countries such as South Africa (SA) that have an extensive Gene Xpert infrastructure, and the technology lends itself to onsite point-of-care testing using portable Xpert platforms such as Xpert Edge.

While RT-PCR currently remains the imperfect gold standard for the rapid confirmation of SARS-CoV-2 infection, ongoing genetic evolution of the virus highlights the need to closely monitor and review the methodology based on emerging data. It is possible that a better stage-specific reference standard may emerge incorporating immunoassay results.

Limited testing capacity remains a challenge to widespread surveillance and testing in SA. Expanding testing services to research-based laboratories is fraught with challenges, including the need for accreditation of laboratories (by the South African National Accreditation System in SA) and personnel (by the Health Professions Council in SA). Capacity shortfalls are further compounded by an international and countrywide shortage of kits and reagents and a severe reduction in international freight shipping capacity. However, the implementation of rapid automated molecular testing (Xpert Xpress SARS-CoV-2) will be helpful if enough cartridges can be procured.

## Immunoassays and their utility

Several antigen-based immunoassays have been developed that detect antibodies in serum or plasma.^[30]^ One such assay was recently FDA approved, and the Foundation for Innovative New Diagnostics website lists over 200 companies that are either making or have made such assays.^[31]^ Some are rapid lateral flow assay (LFA)-based while others are enzyme-linked immunosorbent assay (ELISA)-like tests. Both formats have antigen impregnated on a test line or on a plastic plate surface, and detect human IgG or IgM, and sometimes also IgA antibodies. In the meantime, rapid capture assays that detect viral antigens in nasopharyngeal aspirates have also been developed, and are being evaluated in tandem.

Despite the proliferation of different testing devices and kits that are emerging, there are very few independent validation data on which specific assays work optimally. Therefore the sensitivity, specificity and predictive values of individual tests in different contexts remain unknown. A web-based resource has been developed that lists assays that have now been approved for use in specific countries.^[32]^ Some tests purchased by specific countries have already been found not to meet expectations.^[33]^ In Spain, one of worst-hit European nations, health authorities purchased thousands of rapid serological tests from a biotech company in China, but these were later found to have a sensitivity of ~30%.^[34]^ SA companies have already produced iterations of LFA platforms, and they are currently being evaluated.

One concern is test specificity, as there are four common human coronaviruses that cause up to a third of common cold episodes. Poorly designed antibody tests may cross-react with pre-existing anti-coronavirus antibodies. High false-positivity rates may erroneously indicate disease in those without COVID-19, resulting in wasted public health contact-tracing efforts, unnecessary anxiety and even worse, unintended exposure of individuals to COVID-19 in testing centres and wards if they are hospitalised. Sub-optimal sensitivity with LFA formats without a signal amplification step is a potential concern as LFA, depending on the context, may have suboptimal sensitivity compared with ELISA-based assays. However, to what extent this applies to COVID-19 remains to be seen.

Several recent articles describe longitudinal antibody responses in patients with COVID-19.^[35–38]^ Broadly speaking, IgM responses tend to become detectable 3 - 7 days after the onset of symptoms.^[37,39]^ Robust responses generally develop during the second week of illness.^[35,36]^ Given these considerations, antibody-based tests are not recommended for first-line diagnosis within the first few days of symptoms. There is some evidence that combining antibody and RT-PCR data during the early phase of disease may be useful and may have some incremental benefit, though further studies are required. However, the SA Health Products Regulatory Agency and other agencies have recently indicated, based on guidance from the SA National Institute of Communicable Diseases and the World Health Organization, that serological testing is not suitable for diagnosis of acute SARS CoV2 infection, and should be limited to epidemiological surveys (at least until more data become available).

Indeed, there is an undisputed role for immunoassays in surveillance studies, which may guide public health planning and help to define the trajectory of the epidemic. Their potential role for targeting vaccination in certain subgroups is being investigated. Some have suggested that immunoassays could identify previously infected and recovered healthcare workers and other essential workers who could potentially return to work, on the assumption that they are immune to reinfection. Whether this is the case, and for how long immunity lasts in the case of COVID-19, remains unclear. Therefore the validity of the concept of ‘immunity passports’ remains unclear.^[40]^

## HIV-COVID-19 co-infected persons

There are currently no data on how diagnostic, management and prognostic considerations may be different in HIV-infected v. uninfected persons. In HIV-infected patients presenting with a respiratory tract infection, the general possibilities outlined above have to be considered, including considering *Pneumocystis carinii* pneumonia and TB in the differential diagnosis. Although well documented, it is not widely appreciated that between 10 and 20% of community-acquired pneumonia or acute lower respiratory tract infection in sub-Saharan Africa and parts of Asia is due to *Mycobacterium tuberculosis*^[41–43]^, and this proportion is even higher in HIV-infected persons.^[41,43]^ It is also possible that COVID-19 infection may unmask subclinical TB in both HIV-infected and uninfected persons. On the other hand, and particularly in HIV-infected persons, COVID-19 pneumonia, as in the case of influenza,^[43]^ may be associated with a poorer prognosis in hospitalised patients with TB. Whether the SARS-CoV-2 viral load will be higher in HIV-infected persons, and therefore RT-PCR sensitivity better, remains unclear. In HIV-uninfected persons, co-infection with more than one pathogen has already been documented, e.g. co-infection with COVID-19 and influenza and/or bacterial organisms.^[44]^ It is possible that co-infection with more than one pathogen may be more frequent in HIV-infected persons or those with advanced immunosuppression. These unanswered questions will only be resolved once more data become available.

## Summary and conclusions

The rapidly spreading COVID-19 pandemic has exposed capacity weaknesses in healthcare and laboratory testing systems. Although the mainstay of testing remains RT-PCR, there are several drawbacks, including a significant false negative rate in the early course of the disease, assay cost and lack of assay simplicity and the requirement for complex laboratory infrastructure. There is an emerging shortage of reagents, including RNA extraction kits, that is likely to worsen; already many centres are using dry nasopharyngeal swabs because of the shortage of viral transport medium. Antibody-based immunoassays have been developed, although they have a limited role in the early diagnosis of symptomatic patients. Their incremental benefit over RT-PCR assays, and their role in other applications, including surveillance and targeting of individuals for vaccination and redeployment into the workforce, are under investigation.
